# ClassifieR 2.0: expanding interactive gene expression-based stratification to prostate and high-grade serous ovarian cancer

**DOI:** 10.1186/s12859-024-05981-6

**Published:** 2024-11-21

**Authors:** Aideen McCabe, Gerard P. Quinn, Suneil Jain, Micheál Ó Dálaigh, Kellie Dean, Ross G. Murphy, Simon S. McDade

**Affiliations:** 1 School of Biochemistry and Cell Biology, University College Cork, Cork, Ireland; 2 The SFI Centre for Research Training in Genomics Data Science, Galway, Ireland; 3 BlokBio, Ormeau Labs, Belfast, Northern Ireland UK; 4 The Patrick G Johnston Centre for Cancer Research, Queen’s University Belfast, Belfast, Northern Ireland UK; 5 Department of Clinical Oncology, Northern Ireland Cancer Centre, Belfast Health and Social Care Trust, Belfast, UK; 6 School of Biological and Chemical Sciences, University of Galway, Galway, Ireland; 7 School of Mathematical and Statistical Sciences, University of Galway, Galway, Ireland; 8 The Centre for Genomic Medicine, Ulster University, Coleraine, Northern Ireland UK

**Keywords:** Molecular classification, Shiny application, High-grade serous ovarian cancer, Prostate cancer, Transcriptomics

## Abstract

**Background:**

Advances in transcriptional profiling methods have enabled the discovery of molecular subtypes within and across traditional tissue-based cancer classifications. Such molecular subgroups hold potential for improving patient outcomes by guiding treatment decisions and revealing physiological distinctions and targetable pathways. Computational methods for stratifying transcriptomic data into molecular subgroups are increasingly abundant. However, assigning samples to these subtypes and other transcriptionally inferred predictions is time-consuming and requires significant bioinformatics expertise. To address this need, we recently reported “ClassifieR,” a flexible, interactive cloud application for the functional annotation of colorectal and breast cancer transcriptomes. Here, we report “ClassifieR 2.0” which introduces additional modules for the molecular subtyping of prostate and high-grade serous ovarian cancer (HGSOC).

**Results:**

ClassifieR 2.0 introduces ClassifieRp and ClassifieRov, two specialised modules specifically designed to address the challenges of prostate and HGSOC molecular classification. ClassifieRp includes sigInfer, a method we developed to infer commercial prognostic prostate gene expression signatures from publicly available gene-lists or indeed any user-uploaded gene-list. ClassifieRov utilizes consensus molecular subtyping methods for HGSOC, including tools like consensusOV, for accurate ovarian cancer stratification. Both modules include functionalities present in the original ClassifieR framework for estimating cellular composition, predicting transcription factor (TF) activity and single sample gene set enrichment analysis (ssGSEA).

**Conclusions:**

ClassifieR 2.0 combines molecular subtyping of prostate cancer and HGSOC with commonly used sample annotation tools in a single, user-friendly platform, allowing scientists without bioinformatics training to explore prostate and HGSOC transcriptional data without the need for extensive bioinformatics knowledge or manual data handling to operate various packages. Our sigInfer method within ClassifieRp enables the inference of commercially available gene signatures for prostate cancer, while ClassifieRov incorporates consensus molecular subtyping for HGSOC. Overall, ClassifieR 2.0 aims to make molecular subtyping more accessible to the wider research community. This is crucial for increased understanding of the molecular heterogeneity of these cancers and developing personalised treatment strategies.

**Supplementary Information:**

The online version contains supplementary material available at 10.1186/s12859-024-05981-6.

## Background

Recent advances in affordable next generation sequencing methods have aided in the identification of distinct molecular subtypes within histopathological classifications of cancer. These molecular subgroups possess distinct biological characteristics and are often associated with patient prognosis. Clinically relevant subgroups have been identified in various cancers including breast, colorectal, pancreatic, gastrointestinal, prostate and ovarian [1–6]. Identification of cancer subtypes holds promise for enhancing patient outcomes by facilitating novel therapeutic development, guiding treatment decisions and elucidating the underlying biological differences among anatomically and/or histologically similar cancers. As a result, there exists numerous computational methods for stratifying transcriptomic data from patient samples into molecularly distinct subgroups. However, researchers aiming to leverage the information offered by molecular stratification require bioinformatics expertise, a resource often lacking in labs without computational assistance. Therefore, there is an increasing need to annotate patient data into molecular subtypes in a user-friendly manner to better understand disease mechanisms and improve treatment outcomes.

ClassifieR, our recent solution for colorectal cancer (CRC) and breast cancer, exemplifies this approach [7]. For CRC, we developed ClassifieRc that facilitates the classification of CRC samples into Consensus Molecular Subtypes (CMS) [2] and Colorectal Intrinsic Subgrouping (CRIS) [8]. Similarly, for breast cancer, ClassifieRb allows for classification of samples into PAM50 molecular subgroups and inference of OncotypeDX risk scores. These tools are freely available and provide a comprehensive annotation of transcriptomic data without requiring extensive bioinformatics expertise. Nevertheless, molecular stratification remains a core problem beyond breast and colorectal cancers. Here, we present ClassifieR 2.0 which has extended the functionality of ClassifieR to prostate cancer and HGSOC.

For prostate cancer, various prognostic gene signatures are available commercially, such as the Decipher test [9], the Prolaris Cell Cycle Progression score [10], and the OncotypeDX prostate cancer assay [11]. These gene signatures have demonstrated clinical utility in stratifying patients into high and low risk groups [12]. Whilst the lists of genes that make up these signatures are published, the commercial nature of these tests require that their methods of producing prognostic scores be locked and not made publicly accessible, posing a significant barrier for their use in research settings. In the case of the OncotypeDX prostate cancer assay, their methods to produce prognostic scores have been published. However, as the signature was developed as a real-time polymerase chain reaction (RT-PCR) assay, their prognostic scores cannot be determined on microarray/RNA-sequencing (RNA-seq) gene expression data. As such, research institutes need methods that can infer prognostic information from these signatures and stratify patients into clinically relevant groups based on the expression of the available gene list.

The molecular subgrouping of HGSOC has also been well investigated, leading to the identification of four biologically distinct molecular subtypes with prognostic relevance, termed immunoreactive, proliferative, differentiated and mesenchymal subtypes [5, 8, 13–20]. To standardise the molecular classification of HGSOC tumours, one group developed a consensus random forest classifier, trained on unanimously classified tumours across multiple methods. This effort yielded an R package that implements this consensus subtyping algorithm and five other previously published algorithms, called consensusOV [5]. Despite these advances however, users still need bioinformatics expertise to utilise this R package. Additionally, subtyping HGSOC samples using bulk RNA-seq data presents significant challenges due to the complex nature of the tumour microenvironment (TME). Bulk RNA-seq captures the collective gene expression from a mixture of cell types within the tumour, including cancer cells, immune cells, and stromal cells, making it difficult to distinguish the gene expression profiles of cancer cells alone [38–40]. To address this challenge, cellular deconvolution methods, such as MCP-counter and xCell, can be applied to estimate the relative abundance of different cell populations, including immune and stromal cells, within the bulk RNA-seq data. However, combining these deconvolution methods with subtyping remains complex and inaccessible for many research labs, highlighting the need for a user-friendly platform to facilitate this integration.

**Fig. 1:**
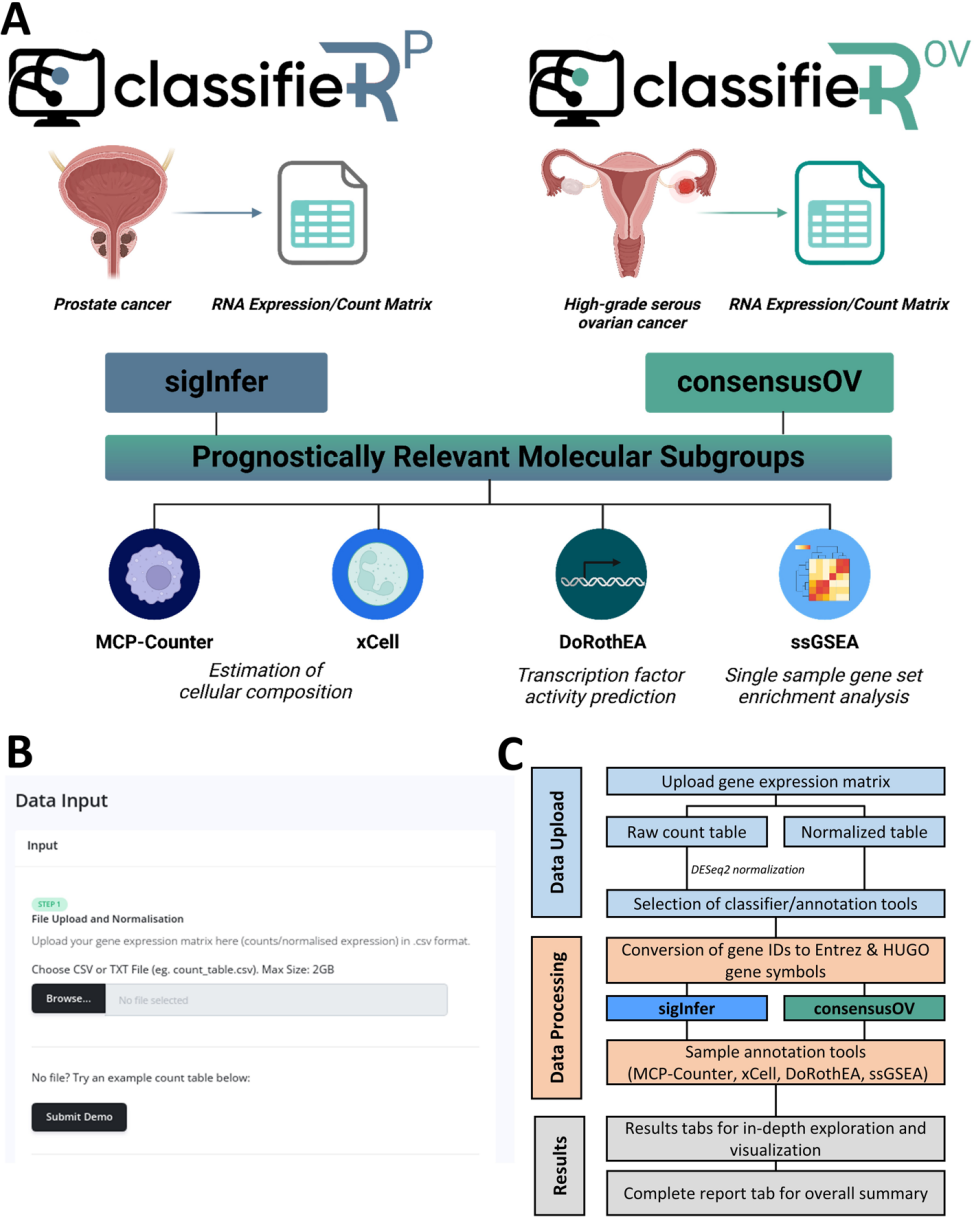
Overview of ClassifieR 2.0. **A.** Visual abstract of ClassifieRp and ClassifieRov. **B.** Screenshot of the graphical user interface (GUI) of ClassifieRp data input page. **C.** Schematic overview of ClassifieRp architecture and sub-functions

To address these issues, we developed ClassifieR 2.0, expanding upon our original framework and introducing key advancements tailored for prostate cancer and HGSOC. ClassifieR 2.0 presents two specialised modules, ClassifieRp and ClassifieRov, dedicated to stratification of prostate cancer and HGSOC samples respectively (Fig. 1). For prostate cancer, ClassifieRp enables the inference of prognostic information from commercial gene signatures (e.g., Decipher, Prolaris), and for HGSOC, ClassifieRov incorporates the consensusOV package to streamline the application of multiple subtyping algorithms. These new modules also retain the functionality of the original ClassifieR framework, including tools for annotating transcriptional subgroups with estimates of cellular composition using Microenvironment Cell Populations-counter (MCP-counter) and xCell, transcription factor (TF) activity predictions using discriminant regulon expression analysis (DoRothEA) and single sample gene set enrichment analysis (ssGSEA; [21–23]).

Our platform is designed to accept input data from multiple transcriptomic technologies, including RNA-seq and microarray, allowing for a broad application in gene expression analysis. Our application also streamlines the workflow by eliminating the need for users to install multiple packages and learn their individual functionalities. By integrating these tools into a single platform, ClassifieR 2.0 simplifies the analysis process, allowing users to apply molecular subtyping and patient stratification methods without requiring detailed bioinformatics expertise or manual data manipulation to utilize different packages. Ultimately, this facilitates a deeper understanding of cancer heterogeneity, supporting improved patient stratification and treatment strategies. Additionally, identifying specific biological pathways or transcription factors enriched in certain subgroups can highlight potential therapeutic targets, guiding the development of targeted therapies tailored to each subgroup.

### Implementation

Similar to ClassifieR [7], ClassifieR 2.0 was developed in an R environment [24] using Shiny [25], enabling the execution of R code within a HTML and JavaScript framework. The application has been orchestrated, hosted, and deployed on a designated CloudCIX Virtual Machine, allowing online access without requiring specific operating systems or additional software. The graphical user interface (GUI) has retained the modern and user-friendly design of ClassifieR, providing detailed instructions on how to use each tool and what information each analysis provides. As with the previous framework, ClassifieR 2.0 can take input from a variety of commonly used transcriptome or array platforms, in the form of a log2 normalised gene expression matrix, a DESeq2 normalised expression matrix [26] or raw gene counts. Upon loading, ClassifieR 2.0 automatically detects whether the data is from RNA-seq or microarray platforms, ensuring compatibility with both technologies. It can process raw RNA-seq reads or microarray intensity data, making the tool accessible to various transcriptomic workflows. Raw RNA-seq reads can be processed to produce these count matrices through accessible web-based platforms such as Galaxy [27]. A demonstrative dataset is also provided to enable users to acquaint themselves with the applications prior to utilisation.

After the data have been uploaded, the user can proceed to choose the classifiers or functional annotation tools to apply to the dataset (sigInfer for ClassifieRp, consensusOV for ClassifieRov, DoRothEA, xCell, MCP-counter and ssGSEA). These packages have undergone internal modifications aimed at enhancing speed of functionality. The resulting molecular classifications are presented in multiple formats, including a summary report, interactive plots and a downloadable CSV table. Functional annotation and interrogation of molecular subgroups can provide valuable insights into the underlying biological pathways and mechanisms associated with each subtype, revealing potential drivers of tumorigenesis. As such, both applications facilitate further functional annotation and interrogation of molecular subgroups. Each analysis yields detailed tabular information and graphical representations, available within each individual tab. Both ClassifieRp and ClassifieRov consolidate outputs from multiple tools into a single downloadable CSV file, merging scores based on sample ID. This allows for interactive visualisation of MCP-counter and DoRothEA transcription factor-activity values within sigInfer/consensusOV transcriptional subgroups.

The sub-applications are accessible at https://classifier.cloudcix.com/classifieRP/ for prostate cancer and https://classifier.cloudcix.com/classifieRov/ for ovarian cancer. Ensuing versions that encapsulate fixes and supplementary features will be rolled out as they are developed.

## Results

Similar to the original ClassifieR framework, ClassifieR 2.0 features a streamlined user interface organised into three main tabs: Introduction, Data Input and Manipulation and Data Output. When the input data has been loaded into the app, automatic detection of whether it has been normalised and which technology it was generated from occurs. As with the previous version, the apps can accept input data from many widely used microarray and RNA-seq platforms. In the case where a certain technology is not available, the user can provide a custom lookup table to facilitate conversion of probe/gene IDs to gene symbol and Entrez IDs, which are utilised by packages within the app. The user can then select the desired analyses from the Settings menu, with the option to select more advanced options if required. After package selection, the user can click the “Classify!” button to run the analyses. Retaining ClassifieR’s ease of use, the classification and annotation of data can be executed without requiring user customization.

In the Processed Data tab, users can access a downloadable expression table, normalised if specified, featuring Gene Symbol identifiers for convenience. Additionally, in the functional annotation tabs (featuring DoRothEA, MCP-Counter and xCell) interactive bar plots, histograms and scatterplots are available to configure and download. These plots were integral to the core functionality of the previous version, illustrating immune cell or transcription factor activities across all samples and enabling users to plot and calculate correlations between two continuous variables. ClassifieR 2.0 integrates cellular deconvolution methods, such as MCP-counter and xCell, directly into the molecular subtyping workflow. These tools estimate the abundance of immune and stromal cell populations from bulk RNA-seq data and provide this information alongside molecular subtyping results.

ClassifieR 2.0 maintains the core functionalities of its predecessor while integrating several additional features. When users input transcriptomic data, ClassifieR 2.0 performs molecular subtyping (e.g., using sigInfer or consensusOV) while simultaneously calculating cell type proportions using cellular deconvolution methods. The results are then visualized through heatmaps and boxplots, allowing researchers to assess the contribution of the tumour microenvironment (TME) to molecular subtypes. This seamless integration enables users to explore TME influences on tumour biology without requiring advanced computational skills. This integrated approach enables detailed annotation of transcriptional subgroups, unveiling critical insights into the underlying biological processes that differentiate these subtypes.

Additional functionalities introduced by ClassifieR 2.0 include the enhancement of heatmaps with column annotations, presenting molecular subgroups for improved interpretability. Moreover, the custom ssGSEA functionality now accommodates Gene Matrix Transposed (GMT) files detailing single gene sets, as this is the typical format provided by databases such as the Molecular Signatures Database (MSigDB). This feature enables users to explore the enrichment of single processes among subgroups via a downloadable boxplot. However, the main additions to the ClassifieR 2.0 framework are the specialised modules; ClassifieRp and ClassifieRov, enabling users to classify prostate and HGSOC transcriptomic datasets respectively.

### ClassifieRp with sigInfer

ClassifieRp enables researchers to infer gene signatures, helping to overcome the financial burden of utilising commercial signatures. It also allows the inference of prognostic groups from gene signatures published without their mathematical models. We also developed sigInfer, a method newly introduced in ClassifieRp which processes input gene expression data by first filtering the dataset to retain only the genes corresponding to the signature of interest. Hierarchical clustering is then used to group patient samples based on expression profiles of these genes. sigInfer offers flexibility in its use, allowing customization of the clustering process through various distance metrics (default: Euclidean) and clustering methods (default: Ward’s method). Users can also adjust the number of patient subgroups (clusters) to be generated (default: two subgroups). In general, prognostic gene signatures generate prognostic scores that are grouped as high or low risk for patients. As such, sigInfer’s default options reflect this by producing two patient subgroups which can be interpreted as high and low risk patients. The output includes sample groupings based on the expression of signature genes, which can be further analysed for prognostic or biological significance. Ultimately, sigInfer’s functionality supports the inference of groups obtained from commercially available gene signatures, such as the Decipher test [9], the Prolaris Cell Cycle Progression score [10], and the OncotypeDX prostate cancer assay [11]. Additionally, sigInfer allows users to input customs gene signatures by uploading their own gene lists.

**Fig. 2:**
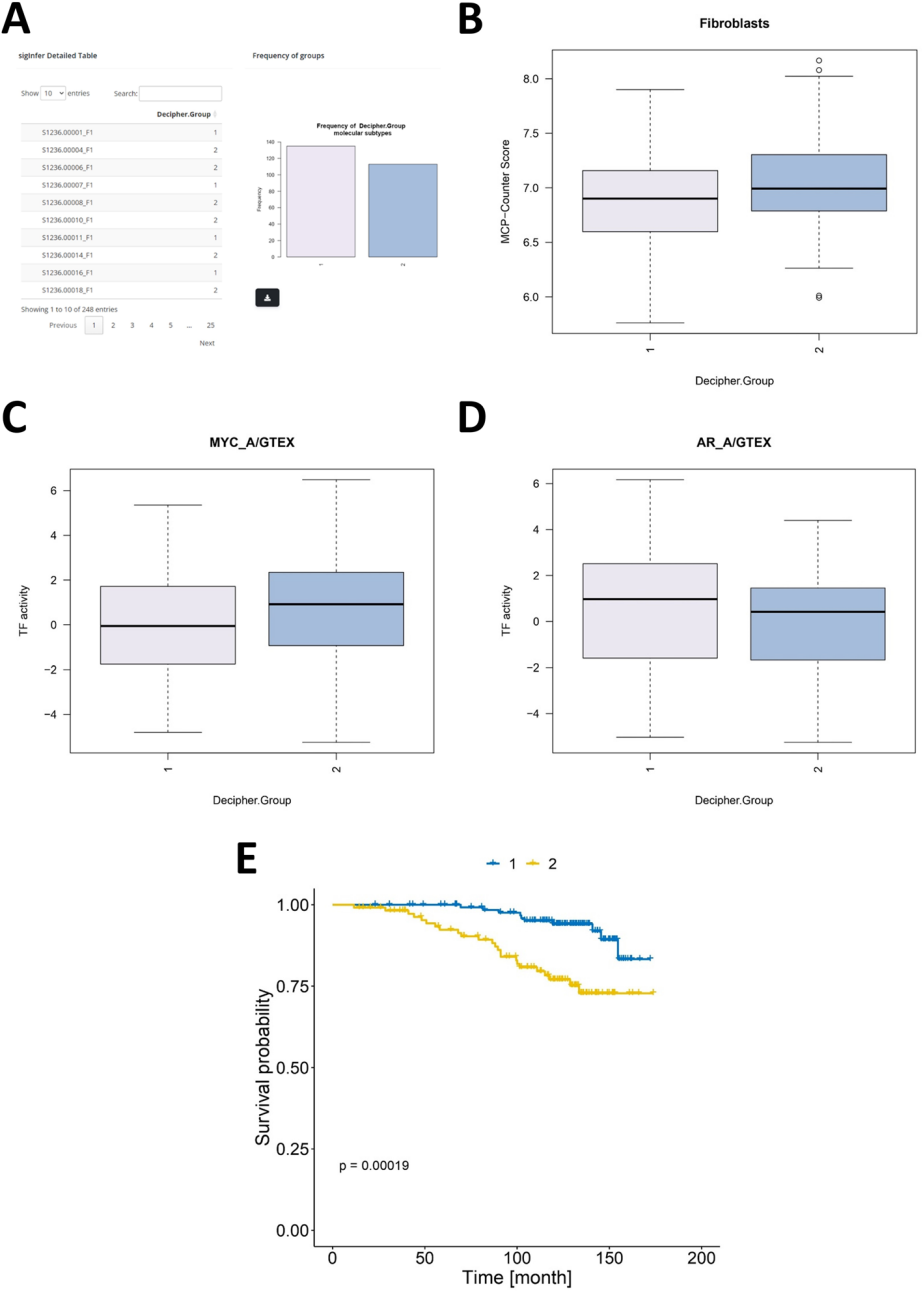
ClassifieRp use case conducted on demo data obtained from prostate cancer gene expression dataset (GSE116918) [29]. **A.** Patient subgroup table and frequency bar plot from sigInfer. **B.** Boxplot of Fibroblast scores from the MCP-counter R package for the patient subgroups 1 and 2 from sigInfer. **C.** Boxplot of MYC TF activity scores from the DoRothEA R package for the patient subgroups 1 and 2 from sigInfer. **D.** Boxplot of androgen receptor (AR) TF activity scores from the DoRothEA R package for the patient subgroups 1 and 2 from sigInfer. **E.** Kaplan–Meier survival curves from the surviveR application for time to metastatic disease of the patient subgroups 1 and 2 from sigInfer

As part of the ClassifieRp module, the sigInfer method was applied to the prostate cancer dataset (GSE116918) using the Decipher test gene signature [9]. The input gene expression data was filtered to retain only the genes corresponding to the Decipher signature, and hierarchical clustering was performed using Ward’s method with Euclidean distance as the metric. Two patient subgroups were identified based on their expression profiles (Fig. 2A). Similar to the original ClassifieR framework, cell type classifiers such as MCP-counter and xCell, TF activity classifiers such as DoRothEA, and functional annotation classifiers such as ssGSEA, are performed in conjunction with the applications’ subgrouping method. Interactive boxplots are produced to demonstrate key TF activity and immune and stromal cell type differences between the sigInfer patient subgroups. By inferring the Decipher prognostic gene signature in the prostate cancer dataset (GSE116918), differences in fibroblast cells (Fig. 2B), androgen receptor (AR; Fig. 2C), and MYC proto-oncogene (*MYC*; Fig. 2D) TF activity between the two patient subgroups are observed. Cancer-associated fibroblast infiltration has been associated with disease progression in prostate cancer [30], whilst high MYC TF activity induces low AR TF activity to drive disease progression and castration resistance in prostate cancer [31]. The sigInfer patient subgroups can be easily integrated with patient-matched survival probability information to be used with the surviveR application [32] for investigating the prognostic potential of the patient subgroups (Fig. 2E). This demonstrates sigInfer's capacity to generate meaningful patient subgroups based on signature expression data and highlights its utility in research settings where commercial prognostic tools may not be accessible.

### ClassifieRov with consensusOV

The ClassifieRov application facilitates the rapid, single-sample classification of HGSOC transcriptional profiles using a selection of classifiers. The default classification method is consensusOV, a consensus random forest classifier trained on unanimously classified tumours across multiple methods, developed by Chen et al*.* [5]. The user also has the option of classification using four other methods published previously [15, 17, 19, 20] using the functionality of the consensusOV R package within the intuitive GUI. The ‘Helland’, ‘Verhaak’ and ‘Konecny’ classifiers can assign subtype scores to each sample based on subtype-specific linear coefficients, subtype-specific ssGSEA, and nearest-centroids with Spearman’s rho respectively [15, 19, 20]. The ‘Bentink’ classifier assigns an angiogenic and non-angiogenic probability score to each sample using the genefu package [17, 36]. Once the chosen classifiers are selected, ClassifieRov applies DESeq2 normalisation to the count matrix, if normalisation has not already been performed, preparing it for utilisation within the consensusOV package.

Upon accessing the Subgrouping tab, users are presented with a comprehensive table showcasing subtype confidence scores assigned to each sample, alongside their respective subtypes (Additional File 1A). Additionally, a simplified downloadable table containing only sample names and subtypes is provided. Furthermore, a bar plot illustrates the frequency distribution of molecular subtypes (Additional File 1B).

**Fig. 3:**
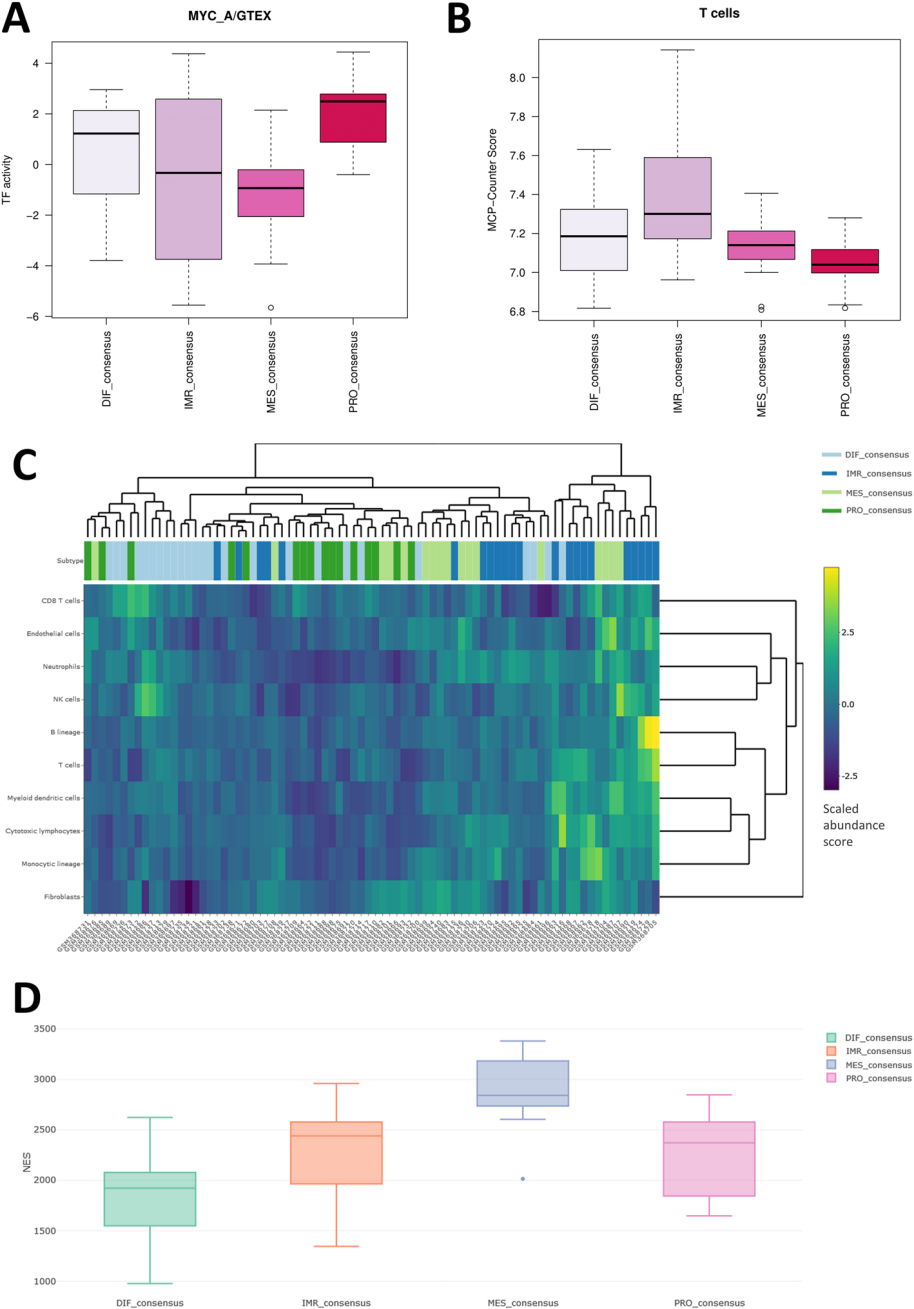
ClassifieRov use case conducted on demo data obtained from GSE14764 [37]. **A.** Interactive boxplot from the Complete Report tab showing distribution of MYC TF-activity scores amongst consensusOV molecular subgroups. **B.** Interactive boxplot from the Complete Report tab showing distribution of MCP-Counter scores for T cells amongst consensusOV molecular subgroups. **C.** Updated heatmap with sample annotations for MCP-Counter scores. **D.** Boxplots showing enrichment score distribution of the MSigDB epithelial-to-mesenchymal transition signature obtained from MSigDB across molecular subtypes. DIF_consensus (differentiated), IMR_consensus (immunoreactive), MES_consensus (mesenchymal) and PRO_consensus (proliferative)

The Complete Report tab aggregates data from all selected classifiers into a downloadable table. Additionally, it features two interactive box plots, enabling visualisation of distinct transcription factor or cell type abundances across molecular subtypes. Here we show increased TF activity of MYC (Fig. 3A), a commonly amplified TF in HGSOC responsible for promotion of uncontrolled cellular proliferation in the proliferative subtype of ovarian cancer [34]. As anticipated, we also observe elevated MCP-Counter score for T cells in the immunoreactive subtype (Fig. 3B), aligning with the expected heightened immune cell infiltration in this subtype [13, 16, 18–20, 35]. Additionally, estimates of immune and stromal cell populations generated using MCP-counter for each tumour sample are displayed as a heatmap, with subtype assignments represented as column annotations (Fig. 3C). The heatmap visualizes clustering of samples based on their gene expression profiles, while integrating cell composition, offering a comprehensive view of the tumour microenvironment's contribution to each subtype. Finally, users can functionally annotate molecular subgroups using ssGSEA. Here we assessed the enrichment of the MSigDB epithelial-to-mesenchymal (EMT) transition signature across molecular subtypes (Fig. 3D). We observed that the mesenchymal subtype exhibited the highest enrichment, indicating a strong association between this subtype and EMT, which has been observed previously [36]. As with ClassifieR, all plots and tables are downloadable, allowing for further post-ClassifieR 2.0 analysis if deemed necessary.

The integration of consensusOV with cellular deconvolution analysis is particularly important for HGSOC due to the heterogeneity of the TME. Recent studies utilising single cell RNA-seq have highlighted how the TME influences subtype assignment [38–40]. For example, the immunoreactive subtype is largely driven by the presence of immune cells, namely macrophages, whereas the mesenchymal subtype is associated with high fibroblast content [38–40]. These subtypes often reflect the influence of non-cancerous cells, which can obscure the transcriptional programmes of cancer cells themselves [40]. In contrast, cancer/epithelial cells typically exhibit either a differentiated or proliferative programme of gene expression program [38–40]. Without incorporating the broader cellular context provided by deconvolution methods, subtyping based on bulk RNA-seq alone may lead to ambiguous interpretations. ClassifieRov integrates tools like MCP-Counter and xCell, enabling users to better interpret the heterogeneity within HGSOC tumours. By integrating cellular deconvolution with molecular subtyping, researchers can more accurately identify whether a subtype's expression pattern is driven by cancer cells themselves or by the tumour microenvironment, thus refining subtype classification and improving the biological relevance of the findings.

## Conclusion

The introduction of ClassifieRp and ClassifieRov addresses the critical issue of accessibility faced by researchers when stratifying their transcriptomic datasets. These tools eliminate the need for specialised bioinformatics expertise to streamline the process of molecular classification and functional annotation for two pervasive diseases. In comparison to existing tools, ClassifieR 2.0 offers an integrated environment where researchers can not only infer established gene signatures but also venture into exploratory analysis by incorporating custom gene signatures. This versatility is further enhanced by the inclusion of methods for immune and stromal cell type estimation, pathway analysis, and transcription factor activity assessment, making it a comprehensive suite for molecular analysis.

Available freely at https://classifier.cloudcix.com/classifieRP/ and https://classifier.cloudcix.com/classifieRov/, the user-friendly interface allows researchers to further functional insights within their datasets, decipher patient prognosis and predict responses to therapy. As with the original framework, ClassifieR 2.0 extends accessibility to tools typically restricted to bioinformaticians, facilitating quicker and concurrent analyses compared to utilising standalone tools. Ultimately, ClassifieR 2.0 aims to expedite the integration of molecular profiling into the clinic, which is crucial for precision oncology and medicine.

## Supplementary Information

Additional file 1: ClassifieRov use case conducted on demo data obtained from GSE14764: Supplementary Images. A: Detailed classification table with subtype scores for each of the four subtypes: DIF_consensus (differentiated), IMR_consensus (immunoreactive), MES_consensus (mesenchymal) and PRO_consensus (proliferative). B: Barplot displaying subgroup frequency and simplified classification table.

## Data Availability

The datasets analysed during this study are available via the Gene Expression Omnibus under the accessions GSE14764 and GSE116918 (https://www.ncbi.nlm.nih.gov/geo/query/acc.cgi?acc=GSE14764; https://www.ncbi.nlm.nih.gov/geo/query/acc.cgi?acc=GSE116918).

## Abbreviations

AR: Androgen receptor
CRC: Colorectal cancer
CRIS: Colorectal intrinsic subtypes
CMS: Consensus molecular subtypes
DoRothEA: Discriminant regulon expression analysis
GMT: Gene matrix transposed
GUI: Graphical user interface
HGSOC: High-grade serous ovarian cancer
MSigDB: Molecular signatures database
MCP: Microenvironment cell population
MYC: MYC proto-oncogene
RNA-seq: RNA-sequencing
RT-PCR: Real-time polymerase chain reaction
ssGSEA: Single sample gene set enrichment analysis
TF: Transcription factor
TME: Tumour microenvironment
