# Supplementary figures and images for: ClassifieR 2.0: expanding interactive gene expression-based stratification to prostate and high-grade serous ovarian cancer
Source: BMC Bioinformatics. 2024 Nov 21;25:362. doi: 10.1186/s12859-024-05981-6 (PMC11580654; doi:10.1186/s12859-024-05981-6)

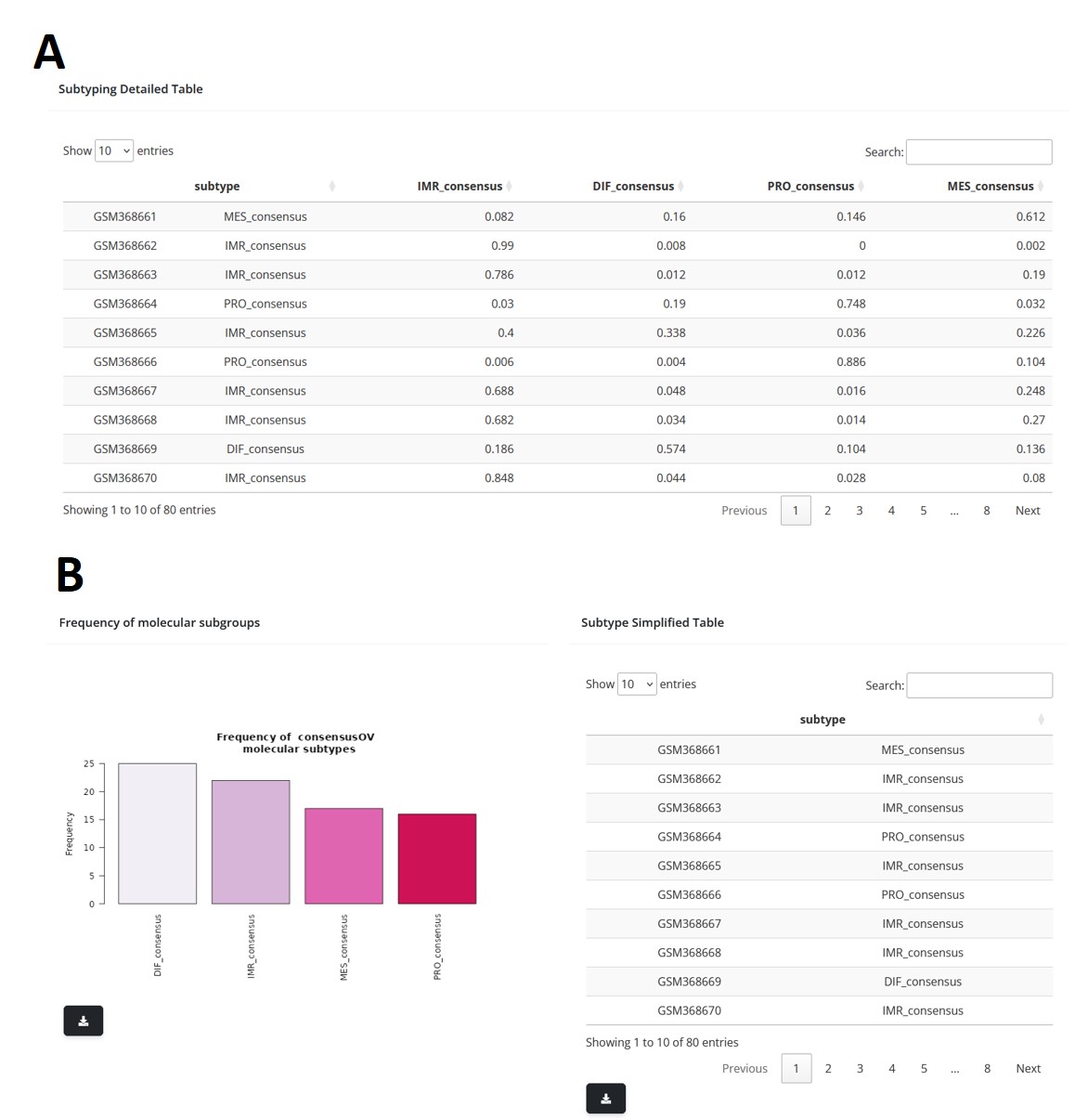

Supplement: Supplementary file 1 — Additional file 1: ClassifieRov use case conducted on demo data obtained from GSE14764: Supplementary Images. A: Detailed classification table with subtype scores for each of the four subtypes: DIF_consensus (differentiated), IMR_consensus (immunoreactive), MES_consensus (mesenchymal) and PRO_consensus (proliferative). B: Barplot displaying subgroup frequency and simplified classification table. [file 12859_2024_5981_MOESM1_ESM.jpg]
